# Modeling and Robust Tracking Decoupling Control of a Coaxial Unmanned Helicopter Based on the Improved Alternating Direction Method of Multipliers

**DOI:** 10.1155/2022/3647784

**Published:** 2022-09-26

**Authors:** Anan Xu, Fang Wang, Ming Chen

**Affiliations:** ^1^School of Aeronautic Science and Engineering, Beihang University, Beijing 100191, China; ^2^Institute of Unmanned System, Beihang University, Beijing 100191, China

## Abstract

In this paper, a robust tracking control strategy based on the dynamic feedback linearization method is proposed for the nonlinear and highly coupled dynamic characteristics of coaxial unmanned helicopter. The mathematical model of the coaxial unmanned helicopter is determined by fault analysis. Then the high-order state system is dynamically feedback linearized by extending the state variables, and the dynamic characteristics of the zeros are analyzed according to the expected tracking characteristics of the inner loop. The pole placement of the subsystem realizes robust monitoring of height and position commands by designing robust compensators. On this basis, an outer loop proportional derivative controller is designed for the horizontal positioning subsystem to realize position tracking. Loop tracking simulation ensures the good separation characteristics of feedback linearization method, and trajectory tracking simulation under fault conditions ensures the control ability and durability of the designed controller.

## 1. Introduction

Under the background of decentralized consensus optimization, composite proxy networks work together on a common decision variable to minimize the sum of their local target activities and limit the information exchange between neighbors. To do this, check the problem first, and then apply Alternating Direction Method of Multipliers (ADMM). In this method, individuals are iteratively calculated and data are exchanged among neighbors. It is found that this method converges rapidly and is considered to be sustainable [1]. For convex optimization problems, we propose an extended Lagrangian method, in which the cost function is the sum of two terms, one of which can be separated into variable blocks and the other lies between continuous variable blocks [2]. Loop is an effective algorithm for solving complex monotone inclusion and convex optimization problems, which consists of many simple blocks. Promoting PRS and ADMM can automatically adapt to the regularity of the problem and provide an improved worst case without convergence regularity. All results are obtained through simple techniques [3]. ADMM is widely used to solve structural cone optimization problems. This paper proves that ADMM is a widely used method for solving large-scale multiblock optimization models, and it can also achieve good convergence [4]. ADMM odds are one of the most effective and successful methods to solve different combinations. Multiblock ADMM is a natural extension of ADMM and a general pattern that is very useful for solving various nonconvex optimization problems. Finally, we propose simulation studies and practical applications to support the correctness of the theoretical statements [5]. In this paper, a sPADMM method is proposed to solve the minimization problem of three-block separable cones, where the second block is the constraint of strong convex functions and connected linear equations (6). In this paper, the control problem of a new rotor/duct fan helicopter is studied. By observing the response of a helicopter to steering input, it is found that the coaxial duct fan has the general hinge characteristics different from traditional helicopters, that is, the strong coupling between ascending and tilting, vertical and rotating motions. The simulation results ensure the correctness of the analysis and control scheme [7]. The design process of strong control of a coaxial micro helicopter is introduced. The process begins with the establishment of a nonlinear dynamic model reflecting all the key elements of a helicopter. Then, the position and climb control controllers are designed using the identified and verified models, and successful flight tests are carried out on the actual system [8]. In this paper, two different methods are used to model a new type of small assembled coaxial helicopter. The system is simulated in two ways and controlled by a PID controller. Simulation results show that the method is effective and can be used to design robust controllers [9]. In this paper, two different methods are used to model a new type of small assembled coaxial helicopter. The system is simulated in two ways and controlled by a PID controller. Simulation results show that the method is effective and can be used to design robust controllers [10]. The initial boundary of closed-loop control of the ideal model of a model autonomous helicopter on the arbitrary flight path is considered. In this paper, a theorem is proved, which sets the limits of the initial fault and trajectory parameters in advance to ensure the acceptable tracking performance of the system. The analysis is expected to be used to validate the work design process [11]. Aiming at the serious nonlinear coupling problem of hydraulic flight simulator, a dynamic robust disconnection compensation method is proposed. This method can disconnect without disconnecting the network. Simulation results show the efficiency of disconnecting the controller. The nonlinear coupling problem in the simulator system is repaired [12]. A robust adaptive fuzzy relative-derived inverse dynamic disengagement control based on the fuzzy linear extended state is proposed and applied to trajectory tracking of the two-degree-of-freedom spherical motion mechanism. The simulation and experimental results ensure the high performance of the controller [13]. Based on the high-dimensional nonlinear system model of the gyro-stabilized platform, a robust control design method for multiple input-output objects is proposed based on eigen structure assignment and quantitative feedback theory. Simulation results show that the EA/QFT method enables designers to understand the design process more intuitively and improves the flexibility between system performance and controller complexity. The results show that even if the spacecraft moves violently, the target tracking system still has good resolution and high-tracking accuracy [14]. The purpose of this paper is to monitor the position and trajectory of a three-stage pneumatic muscle-guided parallel robot without the pressure sensor. An adaptive and durable controller based on the pressure sensor is proposed. The experimental results show that the system not only has good steering accuracy and motion stability but also has strong anti-interference ability [15].

## 2. Unmanned Helicopter Classification

### 2.1. Unmanned Helicopter with the Rotor Tail Rotor

**Figure 1:**
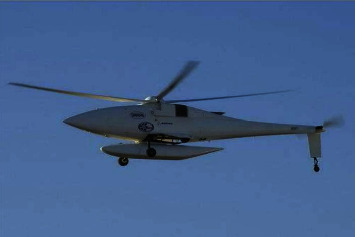
“Hummingbird” A160T unmanned helicopter.

**Figure 2:**
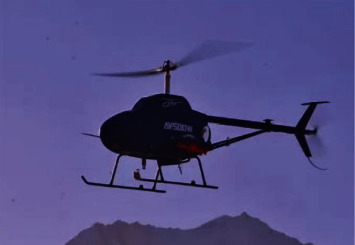
AV500W unmanned helicopter.

The rotor-tail rotor in unmanned helicopter is “rotor + tail rotor,” which is the most common unmanned helicopter type, so it is also called the conventional unmanned helicopter. In the unmanned helicopter with this configuration, a pair of main rotors is used as lifting components on the fuselage, and the rotor traction is adjusted by controlling the common lifting of the main rotors. By adjusting the pitch angle of the main rotor regularly, the inclination angle of the front and rear wings is adjusted, thus controlling the flight of the helicopter. At the same time, a pair of stern rotors is designed in the vertical plane of the tail of the fuselage to balance the reaction torque generated when the rotors turn to the fuselage and realize stability and heading control. The rotor-tail rotor-unmanned helicopter has been proposed, and the related dynamic analysis and flight technology are mature, and the research results are abundant. As shown in Figures 1 and 2, the unmanned Hummingbird A160 T helicopter designed and manufactured by Boeing Company of the United States and the unmanned AV500 W designed and manufactured by China Aviation Industry Corporation both use this assembly design.

### 2.2. Variable Structure Unmanned Helicopter

**Figure 3:**
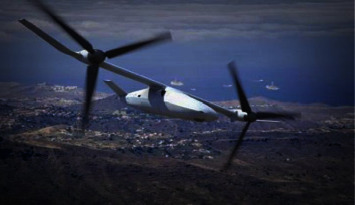
V-247 unmanned tilt-rotor aircraft.

**Figure 4:**
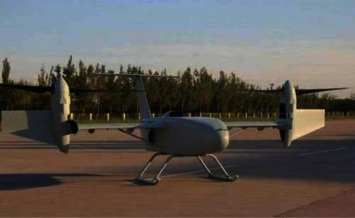
“Rainbow”-10 unmanned tilt-rotor aircraft.

When an unmanned helicopter flies forward at high speed, the supersonic speed of the front tip produces shock wave effect, which greatly increases the aerodynamic drag of the rotor and limits the forward speed of the helicopter. Variable lift unmanned helicopter adopts variable lift axis technology, flies at low speed in helicopter mode, and relies on rotor as lift and steering device to realize short-distance or vertical take-off. By changing the direction of rotor rotation axis to fixed-wing flight mode, the flight speed is gradually increased, which avoids the problems of supersonic overspeed and rotor vibration, and thus improves the forward flight speed. Improved from unmanned helicopters. However, this kind of unmanned helicopter has both aerodynamic characteristics of helicopter and fixed-wing aircraft, and its flight mode is complex. The aerodynamic characteristics of each mode are quite different, and its stability is poor, which increases the design difficulty. Flight control system and flight stability. As shown in Figure 3, only the V-22 Osprey tilt-rotor aircraft and V-22 unmanned V-247 tilt-rotor aircraft of Bell Helicopter Company of America were introduced. At present, China has also done a lot of research on tilt-rotor UAV.  Figure 4 shows the first self-developed tilt-rotor UAV “Rainbow”-10 displayed in Zhuhai Flight Exhibition 2018.

### 2.3. Coaxial Unmanned Helicopter

**Figure 5:**
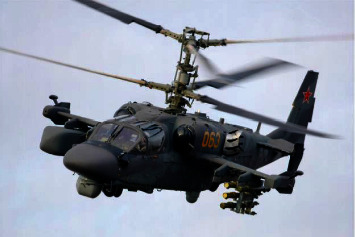
Ka-52 armed helicopter.

**Figure 6:**
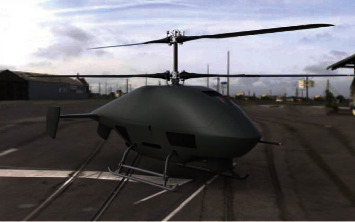
TD450 coaxial unmanned helicopter.

A coaxial unmanned helicopter has two upper and lower rotors rotating in opposite directions, and the direction is controlled by manipulating the torque difference between the upper and lower rotors. The coaxial unmanned helicopter cancels the design and transmission structure of the stern machine, eliminates stern machine failure caused by the stern swing, reduces the weight of unmanned helicopter, and improves the conversion efficiency between the engine and the rotor elevator. At the same time, the fuselage volume of unmanned helicopter is reduced, the structural load is concentrated on the center of gravity, the moment of inertia of helicopter's rising and tilting is reduced, the steering torque of unmanned helicopter is improved, and the maneuverability of unmanned helicopter is improved.  Figure 5 shows the Russian armed Ka-52 helicopter installed in the Russian Air Force for high-altitude reconnaissance and low-altitude operations.  Figure 6 shows the TD450 coaxial unmanned helicopter developed in China, which is applied in the field of agricultural plant protection.

### 2.4. Unmanned Helicopter with Circular Duct

**Figure 7:**
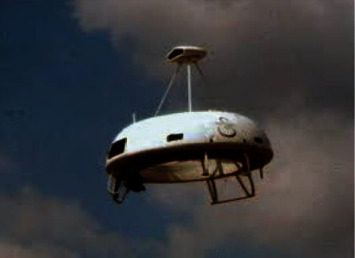
[Password] Unmanned helicopter.

**Figure 8:**
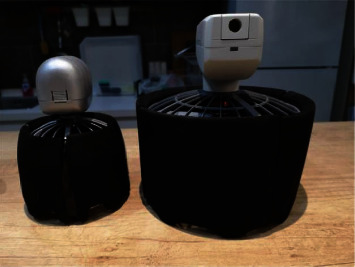
Mini ducted fan drone.

In the annular passage, the unmanned helicopter connects the fuselage with the passage and installs lifting components in the annular passage to reduce the aerodynamic drag of airflow to the rotor during flight. Unmanned helicopters usually use coaxial reversing rotors to balance reverse torque in annular pipes. It is characterized by small overall size and high aerodynamic efficiency at low speed. It is widely used in tactical intelligence, signaling, and other fields. As shown in Figures 7 and 8, the Cypher UAV designed by Sikorsky Company. Unmanned MAV aircraft designed by Honeywell International Company of America and “Golden Eye” produced by Aurora Flight Science Company of America. All UAVs will adopt this structural design.

The unmanned helicopter with a coaxial rotor blower is a new type of unmanned helicopter composed of rotor, duct body, and fan. Different from the annular ducted unmanned helicopter, this helicopter uses the rotor as the main lift device, providing about 70%–80% lift, and the fan in the duct as the auxiliary lift system matches the torque generated by the rotor to realize heading control. The sample unmanned helicopter has the structural characteristics of both the coaxial rotor helicopter and annular ducted helicopter, so it not only retains some aerodynamic characteristics of these two types of helicopters but also has new flight modes and flight control problems that need further study.

## 3. Introduction of the Classical ADMM Algorithm

### 3.1. Brief Introduction of the Development of Classical ADMM Algorithm

#### 3.1.1. Dual Ascending Method

We first give one of the most commonly used models:

(1) $$\begin{array}{c} \min\;f\left(x\right),s.t.Ax=b, \end{array}$$

where *x* ∈ *X* is an optimization variable, *f* : *R*^*n*^⟶*R* ∪ {+*∞*} is a convex function, A is a matrix of *R*^*p*×*n*^, *b* ∈ *R*^*p*^ is a given vector, and *X* is a closed convex set in *R*^*n*^.

The above optimization problem is a linear convex-convex optimization problem. In optimization theory, we do not have algorithms to solve constrained problems directly, but we have many algorithms to solve infinite optimization problems, so our idea is to transform a constrained problem into an unsolvable problem first. The simplest method is the double ascending method. First, according to the constraint condition of binary variable *λ*, the independent variable *X* and binary variable *λ* are updated by using the idea of substitution solution so that the original variable *X* and binary variable *λ* are optimal at the same time. The following is a detailed introduction to the steps of solving the optimization problem by Shuang Li's method. First, the Lagrangian function of the optimization problem is constructed as

(2) $$\begin{array}{c} L\left(x,\lambda \right)=f\left(x\right)-\lambda^{T}\left(Ax-b\right). \end{array}$$

The dual function of formula (2) is given as

(3) $$\begin{array}{c} d\left(\lambda \right)={inf}_{x}\;L\left(x,\lambda \right)=-f^{*}\left(-A^{T}\lambda \right)-b^{T}\lambda , \end{array}$$

where *λ* ∈ *R*^*p*^ is a binary variable. The dual problem is given as

(4) $$\begin{array}{c} \max d\left(\lambda \right), \end{array}$$

where *λ* ∈ *R*^*p*^ also stands for a binary variable. Strong duality assumptions (although minimizing the initial problem min *f*(*x*) is equivalent to maximizing dual problem max*d*(*λ*)) give the same optimal solution to the initial problem as the optimal solution to the dual problem. Assuming that the optimal solutions of the original optimization problem and the double optimization problem are *x*^*∗*^ and *λ*^*∗*^, we have a strong double hypothesis *x*^*∗*^=argmin_*x*_*L*(*x*, *λ*^*∗*^); that is, we can get the optimal solution *x*^*∗*^ of the original problem through the optimal solution *λ*^*∗*^. In the double ascending method, assuming that the dual function *d*(*λ*) is differentiable, its gradient ∇*d*(*λ*) can be estimated by the following method: first, the updated value is obtained, then *d*(*λ*)=inf_*x*_ *L*(*x*^*k*+1^, *λ*)=*f*(*x*^*k*+1^)+*λ*^*T*^(*Ax*^*k*+1^ − *b*), then ∇*d*(*λ*)=*Ax*^*k*+1^ − *b*, which is also called the remainder of the difference of difference constraint *Ax*=*b*. Therefore, the updating process of the Shuang Sheng method is

(5) $$\begin{array}{c} \left\{\begin{array}{c} x^{k+1}≔{argmin}_{x}L\left(x,\lambda^{k}\right)\\ x^{k+1}≔\lambda^{k}+\alpha^{k}\left(Ax^{k+1}-b\right)\text{,} \end{array}\right. \end{array}$$

where *α*^*k*^ > 0 is the step size, and when the step size *α*^*k*^ is selected correctly, the double function *d*(*λ*) rises continuously.

Note that the independent variable *x* ∈ *R*^*n*^ and binary variable *λ* ∈ *R*^*p*^ of the original problem are in different linear states, which means that the double ascending method alternately optimizes in two linear spaces *R*^*n*^ and *R*^*p*^, does not intersect, and develops towards the optimal motion direction. A solution is coming. However, the double slope method also has some disadvantages: if the step size *α*^*k*^ is chosen correctly and the objective function *f*(*x*) is strictly convex and strongly dual, both the independent variable *x*^*k*^ and the binary variable *λ*^*k*^ converge to the optimal point, and the original optimization problem and the double optimization problem can reach the optimal solution time at the same time. Unfortunately, many practical problems can not satisfy the assumptions of strict convexity and strong duality of the objective function *f*(*x*) at the same time, so the Shuang-Li method is useless at this stage.

#### 3.1.2. Dual Decomposition Method

Although the strict conditions of Shuang-Li method are not suitable for solving most practical problems, the Shuang-Li method has a particularly good feature worth exploring. If the objective function *f*(*x*) is separable, we decompose the initial problem into many small problems, optimize these small problems, and then integrate them together to achieve full renewal, which is a double decomposition method. Nevertheless, let us take the optimization problem as an example and look at its decomposition form as

(6) $$\begin{array}{c} \min\;f\left(x\right)=\overset{N}{\underset{i=1}{\sum }}f_{i}\left(x_{i}\right),\mathrm{s.}\mathrm{t.}Ax=\overset{N}{\underset{i=1}{\sum }}A_{i}x_{i}=b, \end{array}$$

where the independent variable is decomposed into *x*=(*x*_1_, *x*_2_,…, *x*_*N*_), *x*_*i*_ ∈ *R*^*n*_*i*_^ is a component of the independent variable *X*, and accordingly the matrix A is decomposed into *A*=(*A*_1_, *A*_2_,…, *A*_*N*_) and satisfies *Ax* ∈ ∑_*i*=1_^*N*^*A*_*i*_*x*_*i*_. Accordingly, the Lagrangian function of formula (6) can be converted into

(7) $$\begin{array}{c} L\left(x,\lambda \right)=\overset{N}{\underset{i=1}{\sum }}L\left(x_{i},\lambda \right)=\overset{N}{\underset{i=1}{\sum }}\left(f_{i}\left(x_{i}\right)+\lambda^{T}A_{i}x_{i}-\frac{1}{N}\lambda^{T}b\right)\text{.} \end{array}$$

In a given iterative process, due to the decomposition of the objective function *f*(*x*), the *x*−part problem of (5) can be divided into the following N-part problems, and optimization is carried out at the same time as

(8) $$\begin{array}{c} \left\{\begin{array}{c} x_{i}^{k+1}≔{argmin}_{x}L_{i}\left(x_{i},\lambda^{k}\right)\\ \lambda^{k+1}≔\lambda^{k}+\alpha^{k}\left(Ax^{k+1}-b\right)\text{.} \end{array}\right. \end{array}$$

It can be seen that in formula (8), decomposition and integration operations are performed at each stage. For subproblems *i* = 1,2,…, *N* and *x*_*i*_−, simultaneous optimization can be realized independently. In the renewal stage of the binary variable *λ*−, we integrate the decomposed constraint residuals *A*_*i*_*x*_*i*_ to form the total residuals *Ax*^*k*+1^ − *b*. Parallel updating of subproblems of the double decomposition method provides theoretical basis and ideas for the following problems.

#### 3.1.3. Augmented Lagrange Multiplier Method

In order to reduce the assumption of the method and improve the applicability and practicability of the method, the following Lagrange coefficient method is added. The solution of adding Lagrange coefficient is to add quadratic addition term (*β*/2)‖*Ax* − *b*‖_2_^2^ to Lagrange function first and the addition delay of formula (1) is obtained as

(9) $$\begin{array}{c} L_{\beta }\left(x,\lambda \right)=f\left(x\right)-\lambda^{T}\left(Ax-b\right)+\frac{\beta }{2}{\left\|Ax-b\right\|}_{2}^{2}, \end{array}$$

where *x* ∈ *X* is still the variable to be optimized, and *λ* ∈ *R*^*p*^ is the Lagrange coefficient. *β* > 0 is the penalty parameter of constraint, which is also called penalty coefficient. Let *β*=0 be the general Lagrangian function *L*_0_(*x*, *λ*) in formula (9), where the additional term *β*/2‖*Ax* − *b*‖_2_^2^ plays a major guiding role in the iterative process because after introducing the added Lagrangian function, the optimal solution of formula (1) is transformed into the minimum solution of the extended Lagrangian function (9), which is far from the feasible range, then *β*/2‖*Ax* − *b*‖_2_^2^ is larger. The minimum solution of infinite problem (*∗*) or the solution of each iteration *x*', *y*' certainly cannot gradually approach the feasible range of the original problem by the limit term *β*/2‖*Ax* − *b*‖_2_^2^. When *x*', *y*' becomes a realizable point, this realizable point *x*', *y*' is the optimal solution of the model. In fact, the extended Lagrangian function *L*_*β*_(*x*, *λ*) can also be equivalent to the general Lagrangian function of the following optimization model in the form:

(10) $$\begin{array}{c} \min\;f\left(x\right)+\frac{\beta }{2}{\left\|Ax-b\right\|}_{2}^{2},s.t.Ax=b. \end{array}$$

Obviously, equality (10) corresponds to equality (1) because for every possible solution *X* in equality (10), there is a penalty term. The dual function formula corresponding to formula (10) of the optimization problem is

(11) $$\begin{array}{c} d_{\beta }\left(\lambda \right)={inf}_{x}L_{\beta }\left(x,\lambda \right). \end{array}$$

The introduction of quadratic penalty term *β*/2‖*Ax* − *b*‖_2_^2^ makes the dual function *d*_*β*_(*λ*) uniformly separable under the most relaxed conditions. The updating steps of solving the model by the Lagrange coefficient method are given as

(12) $$\begin{array}{c} \left\{\begin{array}{c} x^{k+1}≔{argmin}_{x}L_{\beta }\left(x,\lambda^{k}\right)\\ \lambda^{k+1}≔\lambda^{k}+\beta \left(Ax^{k+1}-b\right)\text{.} \end{array}\right. \end{array}$$

The difference between the above formula (12) and Shuang Sheng method is, firstly, the additive Lagrange coefficient method introduces the quadratic addition term *β*/2‖*Ax* − *b*‖_2_^2^; secondly, in the double ascending method of *α*^*k*^, the update step of binary variable *λ* is *E*, which is the variable to be redefined in each iteration. In the Lagrange coefficient method, the update step of *λ* is extended to become *β*, that is, a fixed value. The increased Lagrangian coefficient method makes up for the deficiency of the Shuang Lift method and converges under looser conditions (for example, when the objective function *f*(*x*) does not strictly satisfy the convex attribute). Although the extended Lagrange coefficient method is more applicable, it also makes use of the decomposition property of the objective function *f*(*x*), that is, the additive Lagrange method destroys the parallel solution property of the double-lift method and the double decomposition method. In daily multiplication, when the objective function *f*(*x*) must be decomposable, the enlarged Lagrangian function *L*_*β*_(*x*, *λ*) cannot be decomposed, so it is impossible to optimize several smaller *x*_*i*_− subproblems when solving *x*− subproblem.

Some scholars put forward the precision ADMM algorithm on the basis of the Shuang Lift method, double scattering method, and Lagrange coefficient method, which will be explained in detail in the next section.

### 3.2. Introduction to Classical ADMM Algorithms

#### 3.2.1. Classical ADMM Algorithm

Classical ADMM algorithm inherits the advantages of Shuang Lift method, double scattering method, and Lagrangian coefficient method. Especially, because the objective function *f*(*x*) of optimization problem is complex, the classical ADMM algorithm is particularly prominent in solving such problems. Next, we give the problem model and basic framework of the classical ADMM algorithm.

We consider the following forms of problem optimization:

(13) $$\begin{array}{c} \min_{x,y}\;f\left(x\right)+g\left(y\right),s.t.Ax+By=b, \end{array}$$

where *x* ∈ *X*, *y* ∈ *Y* is the optimization variable and *f* : *R*⟶*R* ∪ {+*∞*}, *g* : *R*^*m*^⟶*R* ∪ {+*∞*} is a convex function, and these initial problems can be transformed into the form of formula (13) after redefining the variables. For example, given *y*=*Ax*, the original problem min*f*(*x*)+*g*(*Ax*) can be transformed into (13), where *B*=−1, *b*=0. In some problems, object functions have good properties such as strictly convex or squared convex functions or having a continuous Lipchitz gradient and so on.

For formula (13), we obtain its augmented Lagrangian function as

(14) $$\begin{array}{c} L_{\beta }\left(x,y,\lambda \right)=f\left(x\right)+g\left(y\right)-\lambda^{T}\left(Ax+By-b\right)+\frac{\beta }{2}{\left\|Ax+By-b\right\|}_{2}^{2} \end{array}$$

The basic idea of the classical ADMM algorithm is to obtain the extended Lagrangian penalty function and then solve the original problem by solving a series of subproblems on *X* and Y and some iterative division as

(15) $$\begin{array}{c} \left\{\begin{array}{c} y^{k+1}≔{argmin}_{y}L_{\beta }\left(x^{k},y,\lambda^{k}\right)\\ x^{k+1}≔{argmin}_{x}L_{\beta }\left(x,y^{k+1},\lambda^{k}\right)\\ \lambda^{k+1}≔\lambda^{k}+\beta \left(Ax^{k+1}+By^{k+1}-b\right)\text{.} \end{array}\right. \end{array}$$

Replace the extended Lagrangian function in (15) with Algorithm 1:

Let *k* = *k*+1 go to step 2.

Compared with the method of adding Lagrange coefficients to update independent variables *x* and *y* simultaneously, each iteration of the classical ADMM algorithm can be divided into the following three steps: the first step is to solve the minimization problem related to updating variable *y*; the second step is to solve the minimization problem related to updating variable *X*; and the third step is to update the Lagrangian multiplier *λ*. In the updating step, *λ* is taken as the extended Lagrangian penalty parameter *β*. The classical ADMM algorithm considers that the objective functions F and *G* which are separated from the independent variable *X*. It allows the algorithm to be divided into two subproblems of a variable and alternately update variables *X* and *Y*, which are the origin of the inverse multiplication name. In addition, in the classical ADMM algorithm, the order of these two subproblems can be arbitrary: first, solve subproblem *y*−, and then solve subproblem *x*−, such as algorithm 1. First, the subproblem can be solved in inverse *x*−. The order of different solutions has no effect on the convergence of the algorithm. Although alternative upgrades require more steps than general upgrades, because it is easier to calculate the two subproblems of alternative upgrades, the classical ADMM converges faster than other methods, so it is more widely used.

#### 3.2.2. Simplified Form of the Classical ADMM Algorithm

Here is another similar classic ADMM form, which is relatively simple and common in practical application fields. Let *r*=*Ax*+*By* − *b*, *u*=1/*βλ* be

(16) $$\begin{array}{c} -\lambda^{T}\left(Ax+By-b\right)+\frac{\beta }{2}{\left\|Ax+By-b\right\|}_{2}^{2}\\ =\lambda^{T}r+\frac{\beta }{2}{\left\|r\right\|}_{2}^{2}=-\frac{\beta }{2}{\left\|r+\frac{1}{\beta }\lambda \right\|}_{2}^{2}+\frac{1}{2\beta }{\left\|\lambda \right\|}_{2}^{2}\\ =-\frac{\beta }{2}{\left\|r+u\right\|}_{2}^{2}+\frac{\beta }{2}{\left\|u\right\|}_{2}^{2}\text{.} \end{array}$$

Accordingly, the augmented Lagrange function is also equivalent to

(17) $$\begin{array}{c} L_{\beta }\left(x,y,\lambda \right)=f\left(x\right)+g\left(y\right)-\lambda^{T}\left(Ax+By-b\right)+\frac{\beta }{2}{\left\|Ax+By-b\right\|}_{2}^{2}\\ =f\left(x\right)+g\left(y\right)--\frac{\beta }{2}{\left\|r+u\right\|}_{2}^{2}+\frac{\beta }{2}{\left\|u\right\|}_{2}^{2}\text{.} \end{array}$$

By the same token, the iteration of the subproblem is

(18) $$\begin{array}{c} \left\{\begin{array}{c} y^{k+1}≔{argmin}_{y}\left(g\left(y\right)+\frac{\beta }{2}{\left\|Ax^{k}+By-b+u^{k}\right\|}_{2}^{2}\right)\\ x^{k+1}≔{argmin}_{x}\left(f\left(x\right)+\frac{\beta }{2}{\left\|Ax+By^{k+1}-b+u^{k}\right\|}_{2}^{2}\right)\\ \lambda^{k+1}≔\lambda^{k}+\beta \left(Ax^{k+1}+By^{k+1}-b\right)\text{.} \end{array}\right. \end{array}$$

The simplified form of formula (18) is exactly the same as the original form of formula (15). The simplified form of ADMM is often used in practical applications, but if we want to emphasize the role of binary variables, we must use the original form of ADMM.

#### 3.2.3. Extended Form of the Classical ADMM Algorithm

If the objective function can be decomposed into *N* parts, the form is

(19) $$\begin{array}{c} \min_{x_{1},x_{2},\ldots ,x_{N}}g_{1}\left(x_{1}\right)+g_{2}\left(x_{2}\right)+\cdots +g_{N}\left(x_{N}\right)\\ s.t.A_{1}x_{1}+A_{2}x_{2}+\cdots +A_{N}x_{N}=b\\ x_{i}\in X_{i},i=1,\cdots ,N\text{,} \end{array}$$

where *g*_*i*_(·), *i*=1,…, *N* is a convex function of *R*^*n*_*i*_^⟶*R*, *x*_*i*_ ∈ *X*, *i*=1,…, *N* is a closed convex set of *R*^*n*_*i*_^, and *A*_*i*_ ∈ *R*^*p*×*n*^, *i*=1,…, *N*, *b* ∈ *R*^*p*^ is a given vector. It is easy to know that the extended Lagrangian function of formula (19) is *L*_*β*_(*x*_1_, *x*_2_,…, *x*_*N*_; *λ*)=∑_*i*=1_^*N*^*g*_*i*_(*x*_*i*_)+*λ*^*T*^(∑_*i*=1_^*N*^*A*_*i*_*x*_*i*_ − *b*)+(*β*/2)‖∑_*i*=1_^*N*^*A*_*i*_*x*_*i*_−*b*‖_2_^2^. Similar to the classical ADMM algorithm, we can get the update steps of the extended ADMM algorithm as

(20) $$\begin{array}{c} x_{1}^{k+1}≔argminL_{\beta }\left(x_{1},x_{2}^{k},\ldots ,x_{N}^{k};\lambda^{k}\right)\\ x_{2}^{k+1}≔argminL_{\beta }\left(x_{1}^{k+1},x_{2},\ldots ,x_{N}^{k};\lambda^{k}\right)\\ \vdots \\ x_{N}^{k+1}≔argminL_{\beta }\left(x_{1}^{k+1},x_{2}^{k+1},\ldots ,x_{N};\lambda^{k}\right)\\ \lambda^{k+1}≔\lambda^{k}+\beta \left(A_{1}x_{1}^{k+1}+A_{2}x_{2}^{k+1}+\cdots +A_{N}x_{N}^{k+1}-b\right)\text{.} \end{array}$$

In recent years, the ADMM algorithm, which can decompose the objective function into several parts or several subblocks, has become a hot research topic for many researchers, and the research results are of great significance. This provides a very powerful method for dealing with large-scale optimization models.

### 3.3. Convergence of the Classical ADMM Algorithm

#### 3.3.1. Convergence of the Classical ADMM Algorithm

A typical model of the classical ADMM algorithm is an optimization problem whose objective function is two distinguishable variables. For the model in this chapter, the convergence of the classical ADMM algorithm has been obtained in many literatures. We use the following two assumptions to prove the convergence of the algorithm:

Suppose 1 function *f* : *R*^*n*^⟶*R* ∪ {+*∞*}, *g* : *R*^*m*^⟶*R* ∪ {+*∞*} is a closed convex function.

The closed convex property of the function of Hypothesis 1 can also be described by the following equivalent property: A function *f*(*x*) satisfies Hypothesis 1 if and only if its graph *epif*(*x*)={(*x*, *λ*)|(*x*, *λ*) ∈ *R*^*n*^ × *R*, *f*(*x*) ≤ *λ*} is a nonempty closed convex set.

Suppose 2 Lagrange function *L*_0_(*x*, *y*, *λ*) has stagnation point.

If Hypothesis 1 holds true, then subtasks *x*− and *y*− have optimal solutions. In addition, suppose 1 can become infinity when the function is nondifferentiable. Although the optimization problem is set under such loose conditions, the solution of its subproblem can still be found. This secondary programming task is obviously solvable. If Hypothesis 2 holds true, then the optimal solution *x*^*∗*^, *y*^*∗*^, *λ*^*∗*^ can be obtained for the two problems mentioned above, and for any *x*, *y*, *λ* satisfying the constraint, the following inequality holds for *L*_0_(*x*^*∗*^, *y*^*∗*^, *λ*) ≤ *L*_0_(*x*^*∗*^, *y*^*∗*^, *λ*^*∗*^) ≤ *L*_0_(*x*, *y*, *λ*^*∗*^). According to assumptions 1 and 2, the Lagrange function *L*_0_(*x*^*∗*^, *y*^*∗*^, *λ*^*∗*^) is limited to each optimal solution *x*^*∗*^, *y*^*∗*^, *λ*^*∗*^. It can be concluded that *x*^*∗*^, *y*^*∗*^ is the solution of (13), so *Ax*^*∗*^+*By*^*∗*^=*b* and *f*(*x*^*∗*^), *g*(*y*^*∗*^) < *∞*. Of course, it can also be concluded that *λ*^*∗*^ is the optimal solution, and we do not make additional assumptions about matrices *A*, *B* and vector *B* in constraints nor do we require matrices A and B to have perfect permutation.

The following will give the convergence result of solving formula (13) with the classical ADMM algorithm:

**Theorem 1 .**

In Hypotheses 1 and 2, the iterative generation sequence of the classical ADMM algorithm satisfies what as follows:

1. When *k*⟶*∞* is the constrained residual set *r*^*k*^=*Ax*^*k*^+*By*^*k*^ − *b*⟶0, the residual converges, that is, the boundary point of the iterative sequence (*x*^*k*^, *y*^*k*^) is the feasible point of formula (13).
2. The objective function is convergent. When *k*⟶*∞*, the sequence *f*(*x*^*k*^)+*g*(*y*^*k*^)⟶*p*^*∗*^; that is, the limit point *p*^*∗*^ of the objective function sequence is the optimal solution of formula (13).
3. When *k*⟶*∞* is a set of bivariate *λ*^*k*^⟶*λ*^*∗*^, the bivariate is convergent; that is, the boundary point of a set of bivariate variables is a double optimum.

Generally speaking, the higher the accuracy of the classical ADMM algorithm, the slower the convergence speed. Especially in practical applications, we faced quite a lot of large optimization problems. If the accuracy is too high, the convergence speed of the classical ADMM algorithm is very slow. If we want to solve this large-scale optimization problem quickly, our method often meets the accuracy requirements of the algorithm and realizes the fast convergence of the algorithm, thus slowly appearing the inaccurate ADMM algorithm.

#### 3.3.2. Optimal Conditions and Stopping Criteria of the Classical ADMM Algorithm

Generally, the optimization problem satisfies the following relationship as

(21) $$\begin{array}{c} \left\{\begin{array}{c} Ax^{*}+By^{*}-b=0\\ 0\in \partial f\left(x^{*}\right)+A^{T}\lambda^{*}\\ 0\in \partial g\left(y^{*}\right)+B^{T}\lambda^{*}\text{,} \end{array}\right. \end{array}$$

and vice versa.

The above formula (21) is called the optimal condition of the classical ADMM algorithm. If *x*^*∗*^, *y*^*∗*^, *λ*^*∗*^ is the optimum of formula (13), then formula (21) is satisfied; on the contrary, if there is *x*^*∗*^, *y*^*∗*^, *λ*^*∗*^ satisfying (21), it is the best point of (13). In equation (21), the first line is called the initial problem feasibility condition; that is, the best point must satisfy the equality constraint of equation (12); the second and third lines are called dual-use conditions, where Operator *∂* represents the subdifferential or subgradient operators that is described in detail in the previous section.

Specific to each iteration, we have formula (22) defined by *x*^*k*+1^

(22) $$\begin{array}{c} 0\in \partial f\left(x^{k+1}\right)+A^{T}\lambda^{k}+\beta A^{T}\left(Ax^{k+1}+By^{k+1}-b\right)\\ =\partial f\left(x^{k+1}\right)+A^{T}\lambda^{k}+\beta A^{T}r^{k+1}\\ =\partial f\left(x^{k+1}\right)+A^{T}\lambda^{k+1}\text{.} \end{array}$$

Likewise, we have formula (23) by definition *y*^*k*+1^=argmin_*y*_*L*_*β*_(*x*^*k*^, *y*, *λ*^*k*^) of *y*^*k*+1^ as

(23) $$\begin{array}{c} 0\in \partial g\left(y^{k+1}\right)+B^{T}\lambda^{k}+\beta B^{T}\left(Ax^{k}+By^{k+1}-b\right)\\ =\partial g\left(y^{k+1}\right)+B^{T}\left(\lambda^{k}+\beta r^{k+1}+\beta A\left(x^{k}-x^{k+1}\right)\right)\\ =\partial g\left(y^{k+1}\right)+B^{T}\lambda^{k+1}+\beta B^{T}A\left(x^{k}-x^{k+1}\right)\text{.} \end{array}$$

The above formula (23) can also be written as the corresponding form *βB*^*T*^*A*(*x*^*k*^ − *x*^*k*+1^) ∈ *∂g*(*y*^*k*+1^)+*B*^*T*^*λ*^*k*+1^, where *βB*^*T*^*A*(*x*^*k*^ − *x*^*k*+1^) is denoted as *s*^*k*+1^, where *s*^*k*+1^ can be considered as a residue (double residue) of the double feasibility condition of formula (21). In formula (22), we still have the remaining *r*^*k*+1^=*Ax*^*k*+1^+*By*^*k*+1^ − *b* for the original feasibility condition.

The simplest stop criterion is request |*f*(*x*^*k*^)+*g*(*y*^*k*^) − *p*^*∗*^| ≤ *ε*, where *p*^*∗*^ represents the optimal solution of the objective function and *ε* is the predetermined precision. But please note that this stop criterion uses a certain value of *p*^*∗*^, and we do not know the value of the optimal solution *p*^*∗*^, so we cannot use this criterion. In practical application, the most commonly used stopping criterion is that when the original residual *r*^*k*^ and the double residual *s*^*k*^ are less than the specified accuracy, the algorithm jumps out of the loop and terminates the iteration as

(24) $$\begin{array}{c} {\left\|r^{k}\right\|}_{2}\leq \varepsilon^{pri},{\left\|s^{k}\right\|}_{2}\leq \varepsilon^{dual} \end{array}$$

where *ε*^*pri*^ > 0, *ε*^*dual*^ > 0 is the accuracy of the principal and dual feasibility conditions.

## 4. Experimental Analyses

### 4.1. Performance Comparison

**Figure 9:**
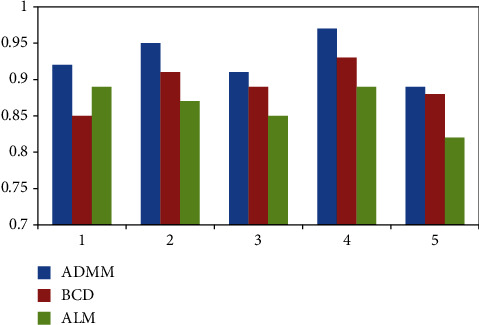
Decoupling ability.

**Figure 10:**
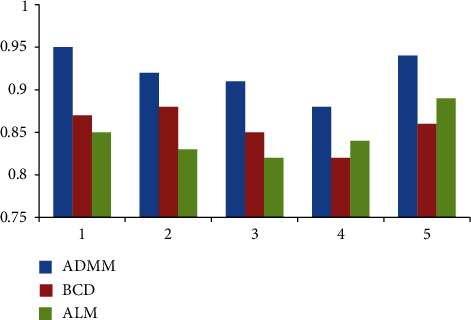
Iterative update capability.

**Figure 11:**
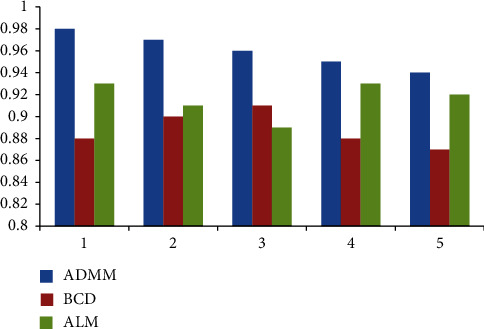
Convergence.

In order to make the modeling of coaxial unmanned helicopter more convenient and accurate, we have done five experiments to compare the performance of ADMM, BCD, and ALM in decoupling ability, iterative updating ability, and convergence. The results are shown in Figures 9–11.

Looking at Figure 9, we can see that in terms of decoupling ability, ADMM is the best, followed by BCD and ALM; From Figure 10, we can conclude that ADMM is the best in terms of iterative update capability, followed by BCD and ALM. From Figure 11, we can conclude that ADMM is the best in terms of iterative update capability, followed by ALM and BCD. Therefore, in order to have higher accuracy in the next experiment, we choose the ADMM method.

### 4.2. Direct Measurement of Physical Parameters

Most parameters can be measured directly, such as weight, length, angle, area, speed, and so on.

The weight of the helicopter rack can be weighed directly, but different tasks have different payloads, and the weight will also change, so consider the center of gravity below.

These directly measurable parameters are displayed in the helicopter assembly group.

It is assumed that all components of France and Italy are on the longitudinal axis of symmetry of the helicopter, so the longitudinal lines intersect, and it is shown in Tables 1–3.

The length of each control arm of the rotor head is measured separately, which is listed in Table 4. By measuring these lengths to determine the value of Bershiller coefficient, compared with other angle measurement methods, the estimation is more accurate.

In this case, gain can be obtained between the inclination changes of the stabilized wing tip as

(25) $$\begin{array}{c} K_{f}=\frac{L_{0}L_{7}}{L_{6}L_{5}}=1.859. \end{array}$$

Because there is no wind tunnel test condition, the frame is approximately a three-dimensional virtual plate, the vertical flow resistance coefficient is about 1, and only the windward side of the body axis is enough in the forward, transverse, and vertical directions. If the helicopter is equipped with shields and other components, the effective aerodynamic drag range within the block can be evaluated according to the relevant discussion of aerodynamic drag of various objects in the references, and it is shown in Table 5.

There are several parameters that are not easy to measure directly but can be estimated from the measured data, as shown in Table 6. The values of *M*_*β*_, *I*_*β*_, and *I*_*β*_^*sb*^ have an important influence on the swinging dynamics, so it is advisable to divide the main blade and stabilizer bar with winglets into several homogeneous parts with regular geometric shapes, integrate them one by one, and then accumulate them to ensure the estimation accuracy. In the table, the dynamic pressure rates *η*_*hs*_ and *η*_*vf*_ of stabilizers are based on experience.

### 4.3. Maneuverability Analysis of the Coaxial Unmanned Helicopter with the Blower

It is known that the coaxial rotor blower-unmanned helicopter has introduced a new aerodynamic structure, and its control mode and motion mode are quite different from those of traditional unmanned helicopters, which need to be studied and analyzed. Therefore, this paper chooses hovering mode as the trim mode and tests the maneuvering and motion characteristics of the unmanned helicopter based on the completely nonlinear mathematical model proposed above.

**Figure 12:**
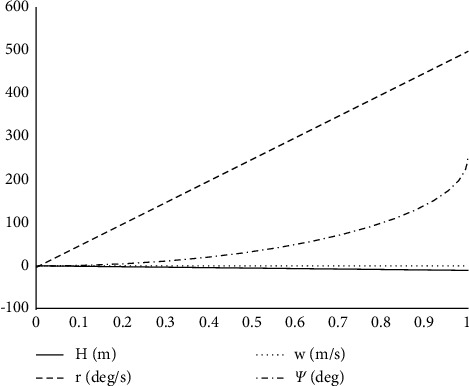
Rotor total pitch step response.

As shown in Figure 12, applying the step signal to the collective rise of the rotor produces a rotational angle, rotational angular velocity, and vertical velocity response. This is due to the increase in the collective rise of the rotor at constant speed, which leads to an increase in lift, which leads to vertical upward acceleration and velocity. Because the vertical downward direction is the positive direction of the *O*_*b*_*Z*_*b*_ axis in the body coordinate system, the vertical velocity response is a burden with the increase of collective ascent. The vertical speed finally approaches a stable value, which indicates that there is a first-order inertia relationship between the rise of the collective rotor and the vertical speed without the influence of fans. At the same time, the increase of collective distance increases the torque of unmanned helicopters. In the balance of reversing torque or external torque of unventilated helicopter, the unmanned helicopter produces steering angle, and the horizontal steering angle responds to directional motion. This shows that the vertical steering input of the unmanned monster helicopter is of course associated with the heading channel.

**Figure 13:**
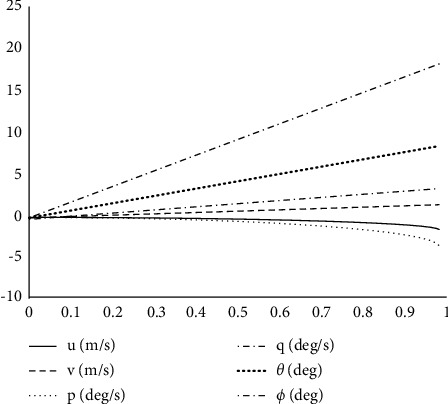
Longitudinal periodic variable pitch step response of the rotor.

**Figure 14:**
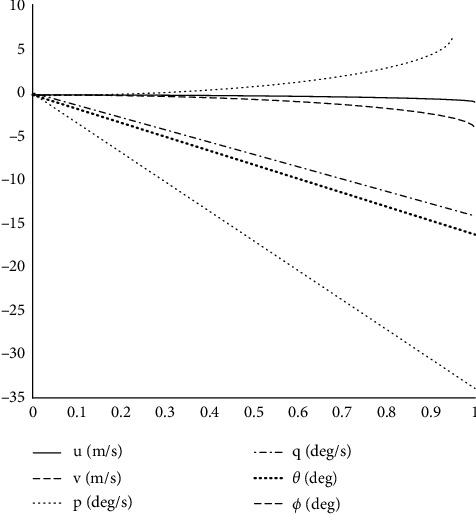
Rotor transverse periodic variable pitch step response.

As shown in Figure 13, the longitudinal periodic pitch signal input of the rotor will cause pitch angle pitch angular velocity and linear velocity response in the *O*_*b*_*X*_*b*_ direction while it will also affect the roll angular velocity roll angle and linear velocity in the *O*_*b*_*Y*_*b*_ direction. The increase of rotor longitudinal periodic pitch leads to positive pitch angular velocity so that the pitch angle increases and the fuselage rises, resulting in the opposite speed to the *O*_*b*_*X*_*b*_ direction. The negative inclination angle reduces the inclination angle, and the frame inclines to the left and sideslip occurs, as shown in Figure 14.

The structure of the unmanned helicopter with the coaxial rotor blower is completely symmetrical, so the unmanned model helicopter is basically the same in the *O*_*b*_*X*_*b*_ direction and *O*_*b*_*Y*_*b*_ direction. The signal input whose distance varies in the longitudinal period has the same dynamic characteristics.

**Figure 15:**
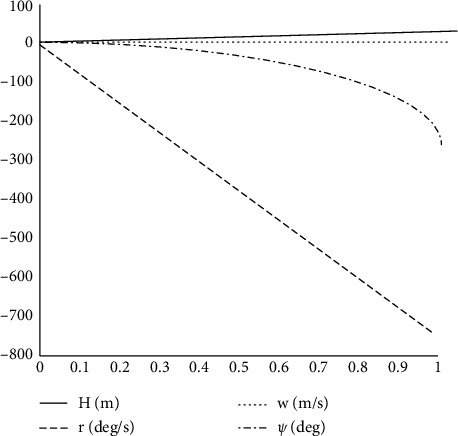
Variable pitch step response of the fan.

**Figure 16:**
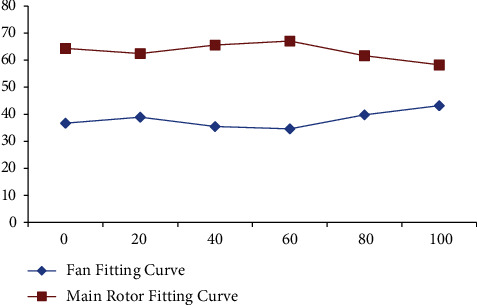
Effect of flight speed on lift distribution.

**Figure 17:**
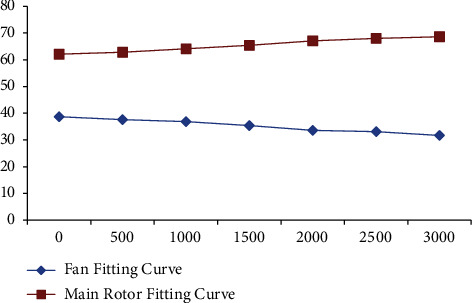
Effect of flight altitude on lift distribution.

As shown in Figure 15, applying a step signal to the fan pitch causes yaw angle, yaw angular rate, and vertical velocity response. Fan pitch change is the input signal of the heading channel, and the fan and rotor in ducted fuselage reverse coaxially. Controlling fan pitch change can offset the antitorque of rotor rotation for fuselage and control the heading motion of the unmanned helicopter through the differential torque between them. Similar to the input signal of the vertical channel, the input signal of the heading channel will also have an impact on the vertical channel, but the effect is smaller than that of the total rotor distance, and it is shown in Figures 16 and 17.

## 5. Concluding Remarks

In this paper, a robust trajectory tracker based on feedback linearization is designed, which is used for the dynamic model of the coaxial unmanned helicopter. Combined with the existing aerodynamic modeling of the coaxial rotor system, a dynamic model which can reflect the aerodynamic disturbance coupling characteristics between coaxial helicopter rotors is established in this paper. In the altitude subsystem, the dynamic feedback linearization of the subsystem is firstly carried out by extending the state variables and analyzing the zero dynamic stability of the system. Then the pole placement of the disconnected subsystem is carried out, and the robust compensator is designed to improve the strength. Finally, the trajectory tracking is realized by designing the outer loop PD control. Simulation results shows that the designed program has good detachment characteristics and still has good tracking performance and robustness in the presence of various model uncertainties.

## Figures and Tables

**Algorithm 1:**
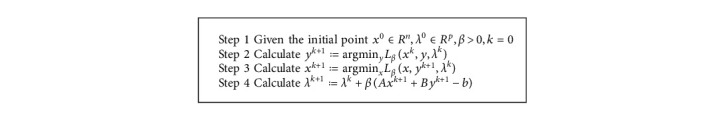
Classical ADMM algorithm standalone.

**Table 1:** Direct measurement parameters of the main smart wing.

Symbol	Physical meaning	Numerical value
*STA*_*h*_	Pulp hub station	0.40 m
*WL*_*h*_	Hub waterline	0.598 m
*WL*_*h*_	Axis anteversion angle	0 rad
Ω	MR angular rate	190.5 rad/s
*m*_*β*_	Main blade weight	0.18 kg
*N*_*b*_	Number of main pulp blades	2
*R*	Plate radius	0.78 m
*c*	Chord length of main pulp	0.059 m
*θ*_*l*_	Blade torsion	0 rad
*K*_*l*_	Pitch-flap coupling rate	0

**Table 2:** Direct measurement parameters of the tail rotor.

Symbol	Physical meaning	Numerical value
*STA*_*tr*_	Tail hub station	1.325 m
*WL*_*tr*_	Tail hub waterline	0.398 m
Ω_*tr*_	TR angular rate	910.1 rad/s
*θ*_*t*_^*tr*^	Blade torsion	0 rad
*N*_*tr*_	Number of tail pulp blades	2
*R*_*tr*_	Plate radius	0.135 m
*c*_*tr*_	Chord length of tail slurry	0.027 m
*k*_*vf*,*tr*_	Double-*v*_*t*_^*tr*^ occlusion factor	0.9

**Table 3:** Direct measurement parameters of the stabilizer bar.

Symbol	Physical meaning	Numerical value (m)
*STA*_*sb*_	SB pulp hub station	0.40
*WL*_*sb*_	SB hub waterline	0.563
*c*_*sb*_	Aileron chord length	0.059
*R*_*n*_	Pulp root separation from pulp hub	0.207
*R*_*n*_	Pulp tip separation from pulp hub	0.312

**Table 4:** Length of each operating stage of the rotor head and Bershiller coefficient.

Symbol	Physical meaning	Numerical value
*L*_0_	SW turntable radius	0.020 m
*L*_1_	MR pitch arm	0.032 m

*L*_2_	Connecting rod of Bershiller mixer	0.015 m
*L*_3_		0.017 m
*K*_*b*_	Bell coefficient	0.322
*L*_4_	Mixing force arm on SB	0.050 m
*L*_5_	SB pitch force arm	0.024 m
*L*_6_	Variable gain connecting rod of SB controlled by the slider	0.013 m
*L*_7_		0.0029 m
*K*_*h*_	Shearer coefficient	0.7324

**Table 5:** Direct measurement parameters of fuselage and stabilizer.

Symbol	Physical meaning	Numerical value
*S*_*x*_^*prj*^	Forward projection area	0.03 m^2^
*S*_*y*_^*prj*^	Lateral projection area	0.11^2^
*S*_*z*_^*prj*^	Vertical projection area	0.06 m^2^
*STA*_*hs*_	HS station	1.035 m
*WL*_*hs*_	HS waterline	0.418 m
*STA*_*vf*_	VF station line	1.280 m
*WL*_*vf*_	VF waterline	0.398 m
*S*_*hs*_	Horizontal tail area	0.017 m^2^
*AR*_*hs*_	Horizontal tail aspect ratio	1.32
*i*_*hs*_	Horizontal tail inclination	0 rad
*S*_*vf*_	Horizontal tail area	0.011 m^2^
*AR*_*vf*_	Vertical tail aspect ratio	3.72
Λ_*vf*_	Vertical tail sweep angle	0.4 rad
*i*_*vf*_	Vertical tail inclination	0 rad

**Table 6:** Re-estimated parameters based on measurements.

Symbol	Physical meaning	Numerical value
*M*_*β*_	Mass moment of main pulp relative to flapping hinge	0.869
*I*_*β*_	Moment of inertia of main pulp around flapping hinge	0.0467
*I*_*β*_^*sb*^	Moment of inertia of auxiliary pulp relative to pulp hub	0.0034
*η*_*hs*_	Effective dynamic pressure rate of horizontal tail	0.8
*η*_*vf*_	Effective dynamic pressure rate of vertical tail	0.9

## Data Availability

The experimental data used to support the findings of this study are available from the corresponding author upon request.
